# Selective Thermotolerant Lactic Acid Bacteria Isolated From Fermented Juice of Epiphytic Lactic Acid Bacteria and Their Effects on Fermentation Quality of Stylo Silages

**DOI:** 10.3389/fmicb.2021.673946

**Published:** 2021-07-26

**Authors:** Nittaya Pitiwittayakul, Smerjai Bureenok, Jan Thomas Schonewille

**Affiliations:** ^1^Department of Agricultural Technology and Environment, Rajamangala University of Technology Isan, Nakhon Ratchasima, Thailand; ^2^Department of Public Health Sciences, Utrecht University, Utrecht, Netherlands

**Keywords:** forage crop, identification, inoculants, isolation, silage fermentation

## Abstract

The aim of the present study was to isolate and identify lactic acid bacteria (LAB) from fermented juice of tropical crops such as Napier grass, Ruzi grass, Purple guinea grass, Stylo legume, and Leucaena and their application to improve the quality of tropical crop silage. Fifteen strains of LAB were isolated. The LAB strains were Gram-positive and catalase-negative bacteria and could be divided into three groups, i.e., *Pediococcus pentosaceus*, *Lactiplantibacillus (para)plantarum*, and *Limosilactobacillus fermentum* according to the biochemical API 50CH test. Based on the analysis of 16S rRNA sequence, the strains isolated in the group *L. (para)plantarum* were distinguished. Two isolates (N3 and G4) were identified as *Lactiplantibacillus plantarum.* Three isolates (St1, St2, and St3) were identified as *L. paraplantarum.* In addition, the identification of other isolates was confirmed in the group *P. pentosaceus* (R1, R4, R5, R8, R11, and L1) and the group *L. fermentum* (N4, G6, G7, and N4). All selected strains were able to grow at 50°C. All LAB strains showed antimicrobial activity against *Escherichia coli* ATCC 25922, *Shigella sonnei* ATCC 25931, *Pseudomonas aeruginosa* ATCC 27853, and *Bacillus cereus* ATCC 11778. Four selected LAB strains (St1, St3, N4, and R4) were tested for their capacity to successfully ensile Stylo legume (*Stylosanthes guianensis* CIAT184). Stylo silages treated with LAB were well preserved, the NH_3_–N and butyric acid contents were lower, and the lactic acid content was higher than those in the control (*p* < 0.05). The acetic acid content was the highest in R4-treated silage among the treatments (*p* < 0.05). The crude protein (CP) content of St1-silage was significantly (*p* < 0.05) higher than the others. The inoculation of thermotolerant LAB selected from fermented juice of epiphytic lactic acid bacteria (FJLB) was found to be highly instrumental to obtain well-preserved silage from the Stylo legume.

## Introduction

Ensiling is a widely used method of preserving moist forage for livestock in many countries. To achieve stable, nutritious silage, a rapid growth of lactic acid bacteria (LAB) is desired because LAB rapidly convert water-soluble carbohydrates (WSC) into lactic acid, thereby causing a rapid decrease in pH, which prevents, among others, excessive proliferation of clostridia (Ni et al., 2015). Naturally occurring epiphytic LAB populations on plant materials are generally heterofermentative and low in initial numbers (Cai, 1999; Eitan et al., 2006), causing a less successful fermentation as indicated by high pH and NH_3_–N content in silage (Liu et al., 2011, 2012). Next to the aformentioned issues, high environmental temperatures may also complicate successful fermentation of forage (Chen et al., 2013; Li et al., 2019). The latter observation may be related to the inability of specific LAB to grow at high temperatures (Weinberg et al., 2001). Indeed, the temperature can reach values up to 50°C (Muck, 2010) when forage is ensiled under tropical conditions. Thus, the inoculation of forage with heat-tolerable LAB prior to ensiling may be instrumental to achieve a well-preserved silage. However, to the best of the author’s knowledge, specific heat-tolerable epiphytic LAB are not yet identified and tested for their potential to successfully ensile tropical forages. In the current study, we isolated and identified LAB from fermented juice of epiphytic LAB (Bureenok et al., 2005) from various tropical forages. Fermented juice of epiphytic LAB (FJLB) contains multiple LAB strains (Wang et al., 2009) and is therefore considered a good source to screen for suitable LAB. Selected LAB were subsequently tested for their capacity to successfully ensile Stylo legume (*S. guianensis* CIAT184). Stylo was selected because of its practical relevance in the tropics.

## Materials and Methods

### Preparation of FJLB and Isolation of LAB Strains

Forage-specific FJLB was prepared from fresh Napier grass (*Pennisetum purpureum*), Ruzi grass (*Brachiaria ruziziensis*), Purple guinea grass (*Panicum maximum* TD58), Stylo legume (*S. guianensis*), and Leucaena (*Leucaena leucocephala*) as described by Bureenok et al. (2005). Briefly, 25 g of each forage was macerated in 50 ml of distilled water in a blender. Then, the content of the blender was filtered over a double layer of sterilized cheese cloths into a glass bottle containing 1% glucose solution. The bottles were capped and stored under anaerobic conditions at 30°C for 3 days. Then, each forage-specific FJLB was spread on lactobacilli de Man, Rogosa, Sharpe (MRS) agar and incubated at 35°C for 48 h under anaerobic conditions. Thereafter, the predominant LAB colony was isolated and purified twice by streaking on MRS agar plates.

### Morphological and Physiological Tests of the Selected Lactic Acid Bacterial Strains

Gram stain, morphology, catalase activity, and gas production from glucose were determined according to the methods for LAB identification as described by Kozaki et al. (1992). Growth at different pH values was observed in MRS broth (adjusting pH with 0.5 N HCl or NaOH) after incubation at 37°C. Growth at different temperatures was observed in MRS broth after incubation at 35°C and 45°C for 5 days. The turbidity of each tube was also noted as an indication of growth or no growth. Each treatment was tested with triplicate tubes. Growth curves for the isolates at 50°C were constructed by plotting the optical density at 600 nm against time. Carbohydrate fermentation was performed by API 50 CHI assay (BioMériux, Marcy-l’Étoile, France). LAB isolates were cultivated in 5 ml of MRS broth overnight at 30°C. The turbidity of the cultured broth was examined by the McFarland method. Cell suspension was transferred into each of the wells on the API 50 CH strips. All wells were coated with sterile liquid paraffin oil and incubated at 30°C. The results were read after 24 h and verified after 48 h. Fermentation of the carbohydrate medium was indicated by a yellow color, except for esculine (dark brown). Color reactions were scored against a chart provided by the manufacturer.

### Identification of LAB Strains by 16S rRNA Sequence Analysis

The DNA of LAB isolates was extracted and purified using a Genomic DNA mini kit (Blood/culture cell) (Geneaid Biotech Ltd., Taiwan) according to the instructions of the manufacturer. Partial fragments of the 16S rRNA genes of each bacterial isolate were amplified using the forward primer 20F (5′-GAG TTT GAT CCT GGC YCA G-3′) and the reverse primer 1500R (5′-GTT ACC TTG TTA CGA CTT-3′) (Brosius et al., 1981). The polymerase chain reaction (PCR) mixtures contained the extracted DNA as a template, 2.0 mM MgCl_2_, 0.2 mM dNTP, and 10 μl of 10X*Taq* buffer, 2.5 units of *Taq* polymerase, and the total volume was brought up to 100 μl. The PCR cycle of reactions consisted of an initial denaturation at 94°C for 3 min followed by 25 cycles of denaturation at 94°C for 1 min, primer annealing at 50°C for 1 min, and primer extension at 72°C for 1 min with a final extension at 72°C for 3 min. The amplicons of LAB were analyzed by means of gel electrophoresis using 0.8% (w/v) agarose and purified with a GenepHlow Gel/PCR Kit (Geneaid Biotech Ltd., Taiwan). The purified PCR products were sequenced by The Macrogen Laboratory (Seoul, South Korea). The resulting 16S rRNA gene sequence of the isolate was analyzed and edited with the use of the Chromas 2.33 and BioEdit program (Hall, 1999). A comparative analysis of 16S rRNA gene sequences from the LAB isolates and all type strains related to the isolate was performed using CLUSTAL W version 1.83 (Thompson et al., 1994). The phylogenetic tree construction based on the 16S rRNA gene was performed using the neighbor-joining approach (Saitou and Nei, 1987) listed in the MEGA version 7 software (Kumar et al., 2016). The phylogenetic distances between the sequences were calculated according to Kimura’s two-parameter model (Kimura, 1980). The robustness of individual branches of the tree was estimated by using bootstrap based on 1,000 replicates (Felsenstein, 1985). The 16S rRNA gene sequence similarities of the isolate were determined using the database of EZBioCloud^1^ (Kim et al., 2012).

### Inhibition Activity Determination of Lactic Acid Bacteria

The isolated LAB strains were inoculated in MRS broth and statically incubated at 30°C for 48 h. Cell-free supernatants were collected by centrifugation (10,000 × *g*, 4°C for 15 min) of LAB cultures and filtered through a 0.22-μm-diameter filter to remove residual cells. The agar well diffusion method (Li et al., 2015) was used to evaluate the antimicrobial activity of the selected LAB strains against the following indicator strains of bacteria: *Escherichia coli* ATCC 25922, *Shigella sonnei* ATCC 25931, *Pseudomonas aeruginosa* ATCC 27853 (Gram-negative bacteria), and *Bacillus cereus* ATCC 11778 (Gram-positive bacteria). Cell-free supernatants (100 μl) were added into wells (7.80 mm in diameter) on nutrient agar plates inoculated with the indicator strains. All plates were incubated for 16–18 h at 30°C. The diameters of inhibition zones were recorded.

### Preparation of the Experimental Silages

Stylo (*S. guianensis* CIAT184) was harvested 60 days after regrowth and chopped with a forage cutter to 2–4 cm and then sampled immediately to determine its macronutrient composition. Four selected LAB strains (*Lactiplantibacillus paraplantarum* St1, *L. paraplantarum* St3, *Limosilactobacillus fermentum* N4, and *Pediococcus pentosaceus* R4) were applied as silage additives. Next to the selected LAB strains, also forage-specific FJLB was prepared from fresh Stylo as previously described. Then, the Stylo was either or not inoculated with 1 × 10^5^ CFU g^–1^ fresh forage of either forage-specific FJLB or one of the selected LAB strains. An equal volume of sterilized distilled water was added to Stylo that was not inoculated with LAB, and this treatment served as a control. Thereafter, ∼100 g fresh material of each experimental forage was tightly packed in oxygen impermeable plastic pouches (20.32 × 33 cm pouches, 120 μm thickness; M-PLASPACK, Bangkok, Thailand), and air was withdrawn from the plastic pouches by a vacuum sealer. Three pouches per treatment were prepared and stored at ambient temperature (37–42°C). After 45 days of ensiling, pouches were opened to assess ensiling characteristics and the macronutrient composition.

### Chemical Analyses

The dry matter (DM) content of fresh Stylo and the experimental forages was determined after oven-drying at 60°C for 48 h. The nitrogen (N) contents were determined by the macro Kjeldahl method (AOAC, 1995). A factor of 6.25 was used to convert N into crude protein (CP). The neutral detergent fiber (NDF) and acid detergent fiber (ADF) contents were determined according to the method of Van Soest et al. (1991), and values are expressed inclusive of residual ash. Buffering capacity and WSC content were determined according to the method as described by Playne and McDonald (1966) and Dubois et al. (1956), respectively.

Lactic acid and volatile fatty acids (VFAs) in silage extracts were measured by HPLC (Aminex HPX-87H, 300 mm × 7.8 mm i.d.; column temperature, 40°C flow rate, 0.60 ml/min, Shimadzu Ltd., Kyoto, Japan). LAB in fresh Stylo legume and the experimental silages were enumerated on MRS agar, and plates were incubated at 35°C for 48 h. The NH_3_–N content of the silage extract was determined using a steam distillation technique (Cai, 2004).

### Statistical Analyses

Data were subjected to one-way analysis of variance (ANOVA); the differences between treatment means were compared using Tukey’s *t*-test using SPSS for Windows version 16.0 SPSS (2007). Statistical Package for the Social Science. SPSS Inc., Chicago. United States. The level of statistical significance was declared at *p* < 0.05.

## Results

### The Morphological and Physiological Properties of LAB Strains Isolated From FJLB

**FIGURE 1:**
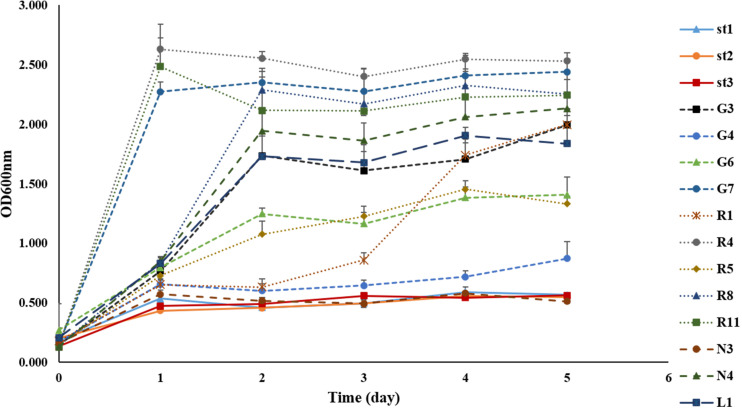
Growth of 15 strains of lactic acid bacteria isolated from forage-specific fermented juice of lactic acid bacteria incubated at 50°C.

The isolation of bacteria using the MRS medium under anaerobic conditions allowed the identification of different LAB with similar or identical morphology from each FJLB. Based on the first step of the screening process, 15 strains were isolated from various forage-specific FJLBs: two strains from Napier grass (N1 and N4), five strains from Ruzi grass (R1, R4, R5, R8, and R11), four strains from Purple guinea grass (G3, G4, G6, and G7), three strains from Stylo legume (St1, St2, and St3), and one strain from Leucaena (L1). All strains were typified as Gram-positive and catalase-negative (Table 1). Among them, R1, R4, R5, R8, R11, and L1 were cocci; others were rod shaped. Based on the end products of glucose fermentation, strains N4, G3, G6, and G7 were classified as heterofermenters, while the remaining strains (N3, G4, St1, St2, St3, R1, R4, R5, R8, R11, and L1) were classified as homofermenters. Except for strain L1 at pH 8, all other strains grew well at various pH levels (3.5, 4.0, 4.5, and 8.0). Moreover, all strains grew well at 35 and 45°C. The ability to grow at 50°C was tested with all the strains (Figure 1) by measuring the density of cell populations in liquid culture over time, and it appeared that the strains St1, St2, St3, N3, and G4 exhibited slight growth, while the other strains grew well.

**TABLE 1:** The characteristics of the selected lactic acid bacteria (LAB) isolates.

	Source of isolated strain of LAB														

	Napier grass		Ruzi grass					Purple guinea grass				Stylo legume			Leucaena
				
LAB strain	N3	N4	R1	R4	R5	R8	R11	G3	G4	G6	G7	St1	St2	St3	L1
Shape	Rod	Rod	Coccus	Coccus	Coccus	Coccus	Coccus	Rod	Rod	Rod	Rod	Rod	Rod	Rod	Coccus
Gram stain	+	+	+	+	+	+	+	+	+	+	+	+	+	+	+
Gas from glucose	**−**	**+**	**−**	**−**	**−**	**−**	**−**	**+**	**−**	**+**	**+**	**−**	**−**	**−**	**−**
Fermentation type	Homo	Hetero	Homo	Homo	Homo	Homo	Homo	Hetero	Homo	Hetero	Hetero	Homo	Homo	Homo	Homo
Catalase activity	**−**	**−**	**−**	**−**	**−**	**−**	**−**	**−**	**−**	**−**	**−**	**−**	**−**	**−**	**−**
Growth at pH															
3.5	+	+	+	+	+	+	+	+	+	+	+	+	+	+	+
4.0	+	+	+	+	+	+	+	+	+	+	+	+	+	+	+
4.5	+	+	+	+	+	+	+	+	+	+	+	+	+	+	+
8.5	+	+	+	+	+	+	+	+	+	+	+	+	+	+	w
Growth at temperature															
35°C	+	+	+	+	+	+	+	+	+	+	+	+	+	+	+
45°C	+	+	+	+	+	+	+	+	+	+	+	+	+	+	+
Identified as (16S rRNA)	*Lactiplantibacillus plantarum*	*Limosilactobacillus fermentum*	*Pediococcus pentosaceus*	*Pediococcus pentosaceus*	*Pediococcus pentosaceus*	*Pediococcus pentosaceus*	*Pediococcus pentosaceus*	*Limosilactobacillus fermentum*	*Lactiplantibacillus plantarum*	*Limosilactobacillus fermentum*	*Limosilactobacillus fermentum*	*Lactiplantibacillus paraplantarum*	*Lactiplantibacillus paraplantarum*	*Lactiplantibacillus paraplantarum*	*Pediococcus pentosaceus*

*+, positive; −, negative; w, weakly positive.*

Based on the API 50CH results, the 15 isolates could be classified into three groups (Supplementary Table 1). Group *P. pentosaceus* consisted of strains R1, R4, R5, R8, and R11 isolated from FJLB of Ruzi grass and strain L1 isolated from FJLB of Leucaena. LAB strains within this group were able to ferment xylose but not D-lactose, D-saccharose, and D-melibiose. Group *L. (para)plantarum* consisted of strain N3 (FJLB of Napier grass), strain G4 (FJLB of Purple guinea grass), and strains St1, St2, and St3 (FJLB of Stylo legume). The LAB strains within the group *L. (para)plantarum* were able to ferment α-methyl-D-mannopyranoside and D-lactose. Group *L. fermentum* contained the strains N4 (FJLB of Napier grass) and G3, G6, and G7 isolated from FJLB of Purple guinea grass. LAB within the group *L. fermentum* produced acid from D-raffinose but not *N*-acetyl glucosamine.

### 16S rRNA Gene Sequencing Analysis

**FIGURE 2:**
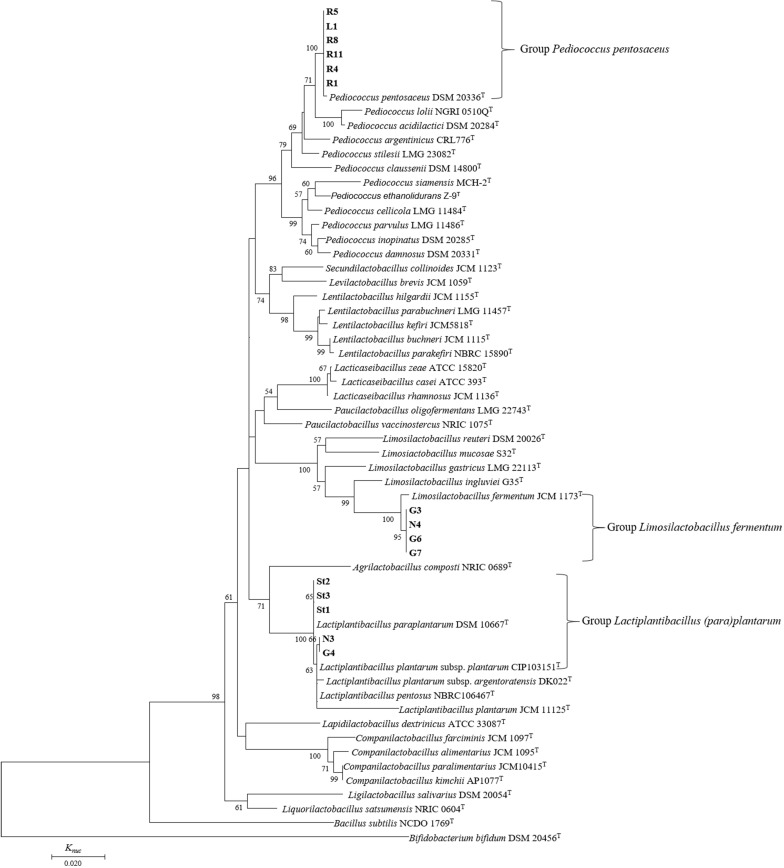
Phylogenetic relationships of lactic acid bacteria isolated from forage-specific fermented juice of lactic acid bacteria. The phylogenetic tree based on 16S rRNA gene sequences of 831 bases was constructed by the neighbor-joining method. Numerals at nodes indicate bootstrap values (%) derived from 1,000 replications. *Bacillus subtilis* NCDO1769^T^ and *Bifidobacterium bifidum* DSM 20456^T^ were used as the outgroup.

In a phylogenetic tree based on 16S rRNA gene sequences, all 15 strains isolated from FJLB were divided into three groups similar to the API analysis (Figure 2). Six strains (R8, R5, R1, L1, R11, and R4) were grouped with *P. pentosaceus* on the phylogenetic tree with a bootstrap value of 100% and showing more than 99% similarity in their 16S rRNA gene sequences. Thus, these strains were identified as *P. pentosaceus*. Considering the phylogenetic positions observed, the type strain of *L. paraplantarum* DSM 10667^T^ was distinguished from *L. plantarum* CIP 103151^T^. Strains N3 and G4 were close to the *L. plantarum* CIP 103151^T^, with 100 and 99.91% similarity in their 16S rRNA gene sequences, respectively. Strains St1, St2, and St3 were categorized in the *L. paraplantarum* cluster and showed a similarity of 16S rRNA of 100% with *L. paraplantarum* DSM 10667^T^. Four strains (N4, G3, G6, and G7) were most closely related to *L. fermentum* JCM 1173^T^. Strains N4, G3, and G6 exhibited 100% 16S rRNA gene sequence pairwise similarities with the closely related species, *L. fermentum* JCM 1173^T^. Only strain G7 showed 99.91% similarity of 16S rRNA gene sequences with the type strain *L. fermentum* JCM 1173^T^.

### Antibacterial Activity Against Pathogenic Bacteria

A total of 15 isolated LAB were tested for antagonistic activity against *E. coli*, *S. sonnei*, *P. aeruginosa*, and *B. cereus* by means of an agar diffusion test. Most of the strains showed antimicrobial activity against *E. coli* and *P. aeruginosa* (Supplementary Table 2). Except for strains G6 and G7, all other LAB strains showed zones of inhibition against *S. sonnei* and *B. cereus*.

### Selected Indices of Fermentation and Chemical Composition of the Experimental Silages

The silage treated with St1 had a higher CP content compared with others (Table 2). N4-silages had lower WSC than the other silages. Lactic acid content was lower in the control silages (*p* < 0.05). The higher amount of acetic acid was higher in silage treated with *L. fermentum* N4. Except for the control silage, butyric acid could not be detected in all the silages treated with either FJLB or selected LAB. Stylo silage inoculated with all LAB strains had lower (*p* < 0.05) NH_3_–N content than the control silage. Control silage pH was higher than the St1-treated silages (*p* < 0.05).

**TABLE 2:** Chemical composition of fresh and ensiled Stylo, and selected indices of fermentation after 45 days of ensiling with or without additional LAB.

	Fresh Stylo	Control	Stylo ensiled with additional LAB					SEM	Sig.
	
			FJLB	LPL-1	LPL-3	LF N4	PP R4		
Chemical composition									
Dry matter (g kg^–1^ fresh)	280	275^ab^	265^ab^	279^a^	258^b^	266^ab^	269^ab^	1.44	0.017
Crude protein	102	90^d^	101^b^	109^a^	98^bc^	101^b^	91^cd^	0.69	<0.001
Neutral detergent fiber	689	673	680	686	649	659	693	6.99	0.487
Acid detergent fiber	499	510^ab^	517^a^	485^ab^	472^b^	497^ab^	514^ab^	3.70	0.026
Hemicellulose	190	163	163	201	177	162	179	7.32	0.631
Water-soluble carbohydrates	45.0	3.0^b^	4.0^ab^	4.8^a^	4.5^a^	1.4^c^	4.1^ab^	0.15	<0.001
Buffer capacity (meq kg^–1^ fresh)	250	na	na	na	na	na	na		
LAB (log CFU g^–1^ fresh)	4.88	6.70^ab^	7.03^a^	6.20^b^	7.15^a^	6.82^a^	7.06^a^	0.05	0.002
Lactic acid	na	33.91^b^	55.54^ab^	75.50^a^	67.60^ab^	86.75^a^	81.72^a^	2.93	0.002
TFa	na	52.91^b^	64.34^b^	85.16^b^	84.43^b^	148.47^a^	89.18^b^	4.12	<0.001
Profile of individual Fa (fraction of TFa)									
LA/TFa	na	0.62^bc^	0.86^a^	0.89^a^	0.83^ab^	0.58^c^	0.91^a^	0.18	<0.001
HAc/TFa	na	0.29^ab^	0.14^bc^	0.11^bc^	0.17^bc^	0.37^a^	0.08^c^	0.02	0.001
HBut/TFa	na	0.06^a^	bdl	bdl	bdl	bdl	bdl	0.005	<0.001
HProp/TFa	na	0.02	0	0	0	0	0.04	0.003	0.093
NH_3_–N (g kg^–1^ total N)	na	121.92^a^	103.64^ab^	70.12^c^	80.64^bc^	84.07^bc^	88.76^bc^	2.82	0.003
pH	5.64	4.71^a^	4.40^ab^	4.32^b^	4.37^ab^	4.41^ab^	4.51^ab^	0.03	0.047

*Unless indicated otherwise, values are expressed as g kg^–1^ dry matter. CFU, colony-forming units; Con, Stylo ensiled without additional lactic acid bacteria; FJLB, fermented juice of epiphytic lactic acid bacteria; LPL-1, *Lactiplantibacillus paraplantarum* St1; LPL-3, *Lactiplantibacillus paraplantarum* St3; LF, *Limosilactobacillus fermentum* N4; PP, *Pediococcus pentosaceus* R4; TFa = total fermentation acids (i.e., lactic acid (LA) + acetic acid (HAc) + propionic acid (HProp) + butyric acid [HBut)]; na, not analyzed; bdl, below detection limit (zero value was used when data were statistically analyzed). Means with different superscripts within rows differ significantly (*p* < 0.05).*

## Discussion

### LAB Strains Isolated From FJLB

Fermented juice of epiphytic LAB has been successfully used as an additive to ensile tropical grasses (Bureenok et al., 2005, 2011). In the current study, homolactic and heterolactic bacteria were isolated from the FJLB of different forage crops, i.e., Napier grass, Ruzi grass, Purple guinea grass, Stylo legume, and Leucaena. Homofermentative LAB produce two molecules of lactic acid from the fermentation of hexoses, whereas heterofermentative LAB produce one molecule of lactic acid, one molecule of other products (acetic acid, propionic acid, or ethanol), and CO_2_ (Kung et al., 2003). In the case of facultative heterofermentation, LAB not only produce mainly lactic acid from hexose but also degrade pentose polymers, such as xylose, to lactic acid and acetic acid or ethanol (Oude Elferink et al., 2000). Based on the results of 16S rRNA analysis, the dominant LAB strains isolated from FJLB were identified as *L. plantarum*, *L. paraplantarum*, *L. fermentum*, and *P. pentosaceus*. Khota et al. (2016) reported that the natural dominant strains of LAB species from Guinea grass and Napier grass were identified as *L. plantarum* and *Lacticaseibacillus casei* that could grow at lower pH and produce more lactic acid compared to the other isolates. Moreover, *L. plantarum* has been isolated from many kinds of grass such as king grass, vetch, tall fescue, and perennial ryegrass (Wang et al., 2017; Shah et al., 2018). The *Pediococcus* spp. have been observed as the prevalent species in forage plants or silages such as corn, Alfafa, Guinea, and Triticale grass (Cai et al., 1999; Kongsan et al., 2019; Soundharrajan et al., 2019). The predominance of LAB species that were found in the silage may be due to the prevalence of Mn^2+^ in plant materials (Daeschel et al., 1987; Boyaval, 1989). Epiphytic LAB such as *L. plantarum*, *L. fermentum*, and *P. pentosaceus* can accumulate Mn^2+^ from plants into their cells, thereby acting as a defense mechanism against oxygen toxicity (Daeschel et al., 1987; Kongsan et al., 2019). In this study, we screened thermotolerant LAB for developing a silage inoculant to be applied under tropical conditions. All LAB strains isolated from FJLB were able to grow at 50°C. Guo et al. (2020) reported that the *L. plantarum* strain isolated from the feces of dairy cows was able to grow at 50°C. Normally, the maximum temperature for optimum LAB growth and reproduction should not exceed 45°C (McDonald et al., 1991). Matsushita et al. (2016) reported that the genomic analysis of thermotolerant strains indicated a large number of mutations that are related to cell surface functions, ion and amino transporters, some transcription factor, and ROS (reactive oxygen species) in cells. There are many reports about LAB strains that have a limited capacity to adapt to high environmental temperatures and therefore have no positive effect on the process of fermentation during ensiling (Chen et al., 2013; Gulfam et al., 2017). Indeed, Guan et al. (2020) reported that LAB could not be detected after 60 days when corn was ensiled at 45°C instead of 30°C. Thus, it seems that thermotolerant LAB strains are potentially of interest to serve as an inoculant to achieve well-preserved silages in (sub)tropical regions. Furthermore, it was observed that most of the isolates in this study were able to inhibit the growth of pathogenic bacteria including *E. coli*, *S. sonnei*, *P. aeruginosa*, and *B. cereus*. Antimicrobial compounds produced by LAB were classified as organic acids, hydrogen peroxide, and bacteriocin-like compound (Heredia-Castro et al., 2015). Li et al. (2015) reported that 39 LAB strains isolated from corn stover silage had inhibitory effect against *Salmonella enterica* ATCC 43971^T^, *E. coli* ATCC 11775^T^, and *Micrococcus luteus* ATCC4698^T^. The current LAB strains have the potential to inhibit the proliferation of undesirable and detrimental microorganisms, which also warrants the use of these LAB in silage making.

### Fermentation and Chemical Composition of the Stylo Silages

Factors such as a low DM and WSC content and a high buffering capacity of material crop indicate poor conditions for lactic acid fermentation (Zhang et al., 2016). Moreover, initial LAB numbers in tropical forages are commonly too low for successful ensiling (Auerbach and Theobald, 2020). The DM content decreased by 0.28% in St1-silage after ensiling. The reduction of the CP content during the fermentation process was because of plant and microbial proteolytic processes in the ensiled material, which change the nitrogenous compounds in silages and result in an increase in soluble N and NH_3_–N (Kung et al., 2018). The low pH values in all LAB-treated silages inhibited the growth of clostridia, which most likely prevented excessive CP loss (Tian et al., 2014). The ADF content of silages treated with St1 and St3 strains was reduced by 2.4–5.8% from the material crop, which is inconsistent with Liu et al. (2012). The decrease in the ADF content indicates a beneficial effect of the treatment in the improvement of the silage nutritive value and probably leads to an increase in silage digestibility in the rumen. It is generally accepted that well-preserved silages should contain pH values less than 4.5 and NH_3_–N content not exceeding 100 g kg^–1^ total N (Kung et al., 2018). In this experiment, four strains of LAB, St1, St3, N4, and R4, were selected to serve as inoculants due to their ability to produce high lactic acid, to grow at a high temperature, to produce antimicrobial activity including the isolation source. In this study, the pH values in all LAB-treated silages were low enough to prevent protein degradation to NH_3_–N. Compared with the homolactic bacteria, heterolactic *L. fermentum* N4 produced greater amounts of acetic acid, but lactic acid was still the predominant end product of fermentation. The current observation is in line with that of Lau and Liong (2014) who also reported that lactic acid was the main acid produced by *L. fermentum*. Despite the greater proportion of acetic acid in the silage treated with *L. fermentum* N4, both the low pH and NH_3_–N values in this silage indicate that Stylo was successfully ensiled when *L. fermentum* N4 was used as an inoculant. The lactic acid content was lower in the control silages. Compared with the homolactic bacteria, heterolactic *L. fermentum* N4 also produced high lactic acid content. *L. fermentum* produced lactic acid in a more predominant amount than acetic acid in MRS broth (Lau and Liong, 2014). This may explain the high production of lactic acid in N4-silage. The higher amount of acetic acid was found when heterolactic *L. fermentum* N4 was added in the silages. Acetic acid is a main fermentation end product when silages are inoculated with heterolactic bacteria (e.g., *Lentilactobacillus buchneri*) with a content approximately 4% DM (Kleinschmit and Kung, 2006). Adding these strains would increase the acetic acid content to inhibit yeast, which could result in better aerobic stability of silages (Paradhipta et al., 2020). The intake of acetic acid (5% as DM basis) did not negatively affect the composition and sensory quality of milk (Daniel et al., 2013); hence, the level of acetic acid in this study will not affect the feed intake and animal performance. Propionic acid is an aerobic microbial inhibitor that can inhibit the activity of molds and yeasts (Chen et al., 2017). The strain of *L. fermentum* under application can increase the aerobic stability of the treated silage. The butyric acid content was higher (*p* < 0.05) in the control silages and not detected in all treated silages. This may be caused by LAB inoculation that reduced the growth of saccharolytic clostridia, which can ferment sugar, lactic acid, and acetic acid to butyric acid (Borreani et al., 2018). N4-silages had lower WSC than the other silages. This may be caused by a greater utilization of WSC to produce a high amount of lactic and acetic acid in this strain. However, low residual WSC content is an important factor for aerobic stability of silage because yeast and molds can utilize WSC resulting in the rapid deterioration of silage after air exposure (Weinberg and Muck, 1996). The fermentation quality of the silages in the current study indicates that the microbial inoculants favorably affected the fermentation of Stylo legume.

## Conclusion

Four selected strains, *L. paraplantarum* St1 and St3, *L. fermentum* N4, and *P. pentosaceus* R4, were shown to improve the fermentation quality and nutritive values of Stylo silage. The silage treated with St1 showed relatively high protein content than control and the other inoculants. The current results suggested that thermotolerant LAB strains isolated from FJLB could be used as a silage inoculant under tropical conditions.

## Data Availability Statement

The datasets presented in this study can be found in online repositories. The names of the repository/repositories and accession number(s) can be found below: https://www. ncbi.nlm.nih.gov/genbank/, MW673709, https://www.ncbi.nlm. nih.gov/genbank/, MW673710, https://www.ncbi.nlm.nih.gov/genbank/, MW673711, https://www.ncbi.nlm.nih.gov/genbank/, MW673712, https://www.ncbi.nlm.nih.gov/genbank/, MW673713, https://www.ncbi.nlm.nih.gov/genbank/, MW673714, https://www.ncbi.nlm.nih.gov/genbank/, MW673715, https://www.ncbi.nlm.nih.gov/genbank/, MW673716, https://www. ncbi.nlm.nih.gov/genbank/, MW673717, https://www.ncbi.nlm. nih.gov/genbank/, MW673718, https://www.ncbi.nlm.nih.gov/genbank/, MW673719, https://www.ncbi.nlm.nih.gov/genbank/, MW673720, https://www.ncbi.nlm.nih.gov/genbank/, MW673721, https://www.ncbi.nlm.nih.gov/genbank/, MW673722, and https://www.ncbi.nlm.nih.gov/genbank/, MW673723.

## Author Contributions

SB, NP, and JS contributed to the conception and design of the study, and wrote sections of the manuscript. NP organized the LAB identifications. SB performed the silage experiment and the statistical analysis. SB and NP wrote the first draft of the manuscript. All authors contributed to manuscript revision, read, and approved the submitted version.

## Conflict of Interest

The authors declare that the research was conducted in the absence of any commercial or financial relationships that could be construed as a potential conflict of interest. The handling editor declared a past co-authorship with the authors.

## Publisher’s Note

All claims expressed in this article are solely those of the authors and do not necessarily represent those of their affiliated organizations, or those of the publisher, the editors and the reviewers. Any product that may be evaluated in this article, or claim that may be made by its manufacturer, is not guaranteed or endorsed by the publisher.

## Footnotes

1 http://eztaxon-e.ezbiocloud.net/

## Supplementary Material

Supplementary Table 1 API 50 CH fermentation patterns of isolated LAB from each FJLB.

Supplementary Table 2 Diameter of inhibition zones caused by the selected strains of LAB which were added to agar plates inoculated with either *Escherichia coli* ATCC 25922 (*E. coli*), Shigella sonnei ATCC 25931 (*S. sonnei*), *Pseudomonas aeruginosa* ATCC 27853 (*P. aeruginosa*), or *Bacillus cereus* ATCC 11778 (*B. cereus*). All values are expressed as mm, mean ± SD.
