# Supplementary material for: Selective Thermotolerant Lactic Acid Bacteria Isolated From Fermented Juice of Epiphytic Lactic Acid Bacteria and Their Effects on Fermentation Quality of Stylo Silages
Source: Front Microbiol. 2021 Jul 26;12:673946. doi: 10.3389/fmicb.2021.673946 (PMC8350162; doi:10.3389/fmicb.2021.673946)
Supplement: Supplementary Table 1 — API 50 CH fermentation patterns of isolated LAB from each FJLB. [file Table_1.DOCX]

**TABLE S1** ׀ API 50 CH fermentation patterns of isolated LAB from each FJLB

|  | *Pediococcus pentosaceus* | | | | | | *Lactiplantibacillus (para)plantarum* | | | | | *Limosilactobacillus fermentum* | | | |
| --- | --- | --- | --- | --- | --- | --- | --- | --- | --- | --- | --- | --- | --- | --- | --- |
|  | R1 | R4 | R5 | R8 | R11 | L1 | N3 | G4 | St1 | St2 | St3 | N4 | G3 | G6 | G7 |
| L-Arabinose | + | + | + | + | + | + | + | + | + | + | + | + | + | + | + |
| Ribose | + | + | + | + | + | + | + | + | + | + | + | + | + | + | + |
| D-Xylose | + | + | + | + | + | + | - | - | - | - | - | - | - | - | - |
| D-Galactose | + | + | + | + | + | + | + | + | + | + | + | + | + | + | + |
| D-Glucose | + | + | + | + | + | + | + | + | + | + | + | + | + | + | + |
| D-Fructose | + | + | + | + | + | + | + | + | + | + | + | + | + | + | + |
| D-Mannose | + | + | + | + | + | + | + | + | + | + | + | - | + | - | - |
| D-Mannitol | - | - | - | - | - | - | + | + | + | + | + | - | + | - | - |
| D-Sorbitol | - | - | - | - | - | - | - | - | + | + | + | - | + | - | - |
| α-Methyl-D-mannopyranoside | - | - | - | - | - | - | + | + | + | + | + | - | + | - | - |
| *N*-acetyl glucosamine | + | + | + | + | + | + | + | + | + | + | + | - | + | - | - |
| Amygdaline | + | + | + | + | + | + | + | + | + | + | + | - | + | - | - |
| Arbutine | + | + | + | + | + | + | + | + | + | + | + | - | + | - | - |
| Salicine | + | + | + | + | + | + | + | + | + | + | + | - | + | - | - |
| D-Cellobiose | + | + | + | + | + | + | + | + | + | + | + | + | + | - | - |
| D-Maltose | + | + | + | + | + | + | + | + | + | + | + | + | + | + | + |
| D-Lactose | - | - | - | - | - | - | + | + | + | + | + | - | + | - | - |
| D-Melibiose | - | - | - | - | - | - | + | + | + | + | + | + | + | + | + |
| D-Saccharose | - | - | - | - | - | - | + | + | + | + | + | + | + | + | + |
| D-Trehalose | + | + | + | + | + | + | + | + | + | + | + | + | + | + | + |
| D-Melezitose | - | - | - | - | - | - | + | + | + | + | + | - | + | - | - |
| D-Raffinose | - | - | - | - | - | - | - | - | + | - | - | + | + | + | + |
| β-Gentiobiose | + | + | + | + | + | + | + | + | + | + | + | + | + | - | - |
| D-Turanose | - | - | - | - | - | - | - | - | - | - | - | - | - | - | - |
| D-Arabitol | - | - | - | - | - | - | - | - | - | - | - | - | - | - | - |
| Gluconate | - | - | - | - | - | - | - | - | - | - | - | - | - | - | - |
| 5-ketogluconate | - | - | - | - | - | - | - | - | - | - | - | - | - | - | - |

+, positive; -, negative; w, weakly positive
