# Supplementary material for: Selective Thermotolerant Lactic Acid Bacteria Isolated From Fermented Juice of Epiphytic Lactic Acid Bacteria and Their Effects on Fermentation Quality of Stylo Silages
Source: Front Microbiol. 2021 Jul 26;12:673946. doi: 10.3389/fmicb.2021.673946 (PMC8350162; doi:10.3389/fmicb.2021.673946)
Supplement: Supplementary Table 2 — Diameter of inhibition zones caused by the selected strains of LAB which were added to agar plates inoculated with either Escherichia coli ATCC 25922 (E. coli), Shigella sonnei ATCC 25931 (S. sonnei), Pseudomonas aeruginosa ATCC 27853 (P. aeruginosa), or Bacillus cereus ATCC 11778 (B. cereus). All values are expressed as mm, mean ± SD. [file Table_2.DOCX]

**TABLE S2** ׀ Diameter of inhibition zones caused by the selected strains of LAB which were added to agar plates inoculated with either *Escherichia coli* ATCC 25922 (*E. coli*), *Shigella sonnei* ATCC 25931 (*S. Sonnei*), *Pseudomonas aeruginosa* ATCC 27853 (*P. aeruginosa*), or *Bacillus cereus* ATCC 11778t (*B. cereus*). All values are expressed as mm, mean ± SD

|  | | *E. coli* | *S. sonnei* | *P. aeruginosa* | *B. cereus* |
| --- | --- | --- | --- | --- | --- |
| *Pediococcus*  *pentosaceus* | R1 | 14.8±0.29 | 12.2±0.29 | 16.2±0.29 | 12.8±1.06 |
|  | R4 | 12.9±1.01 | 15.3±0.58 | 16.8±0.29 | 11.9±0.14 |
|  | R5 | 13.6±0.38 | 15.0±0.00 | 13.0±0.50 | 16.6±0.38 |
|  | R8 | 14.4±0.52 | 14.6±0.14 | 16.7±0.58 | 11.8±0.25 |
|  | R11 | 13.2±0.29 | 14.3±0.58 | 17.2±0.14 | 13.3±0.25 |
|  | L1 | 13.6±0.52 | 16.3±0.43 | 17.1±0.38 | 14.1±0.14 |
| *Lactiplantibacillus (para)plantarum* | N3 | 14.7±0.58 | 15.5±0.87 | 19.2±0.29 | 15.6±0.14 |
|  | G4 | 14.7±0.58 | 15.0±0.00 | 18.8±0.58 | 14.8±0.29 |
|  | St1 | 14.6±0.38 | 15.6±0.14 | 19.5±0.87 | 15.6±0.52 |
|  | St2 | 15.5±0.50 | 16.0±0.00 | 19.2±0.29 | 14.0±0.00 |
|  | St3 | 15.3±0.29 | 16.8±0.25 | 19.1±0.38 | 15.8±0.43 |
| *Limosilactobacillus fermentum* | N4 | 12.3±0.25 | 12.8±0.29 | 16.3±0.58 | 13.4±0.38 |
|  | G3 | 15.8±0.90 | 14.8±0.76 | 17.3±1.04 | 12.0±0.00 |
|  | G6 | 12.8±0.43 | - | 12.8±0.25 | - |
|  | G7 | 12.3±0.14 | 11.7±0.14 | 14.3±0.25 | - |

**-**, no inhibition zone observed
